# Mobile Phone Auscultation Accurately Diagnoses Chronic Obstructive Pulmonary Disease Using Nonlinear Respiratory Biofluid Dynamics

**DOI:** 10.3390/diagnostics15121550

**Published:** 2025-06-18

**Authors:** Caroline Emily Gosser, Luther Daniel, Martin Huecker, Rodrigo Cavallazzi, Hiram Rivas, Jarred Jeremy Thomas, Ryan Close

**Affiliations:** 1 Department of Emergency Medicine, University of Louisville, Louisville, KY 40290, USA; luther.daniel@louisville.edu (L.D.); martin.huecker@louisville.edu (M.H.); jj.thomas@louisville.edu (J.J.T.); 2 Department of Internal Medicine, University of Louisville, Louisville, KY 40290, USA; 3 Center for Interdisciplinary Population and Health Research, MaineHealth, Portland, ME 04102, USA; ryan.close@mainehealth.org

**Keywords:** respiratory sound, chronic obstructive pulmonary disease, auscultation, mobile application, respiratory bio fluid dynamics, mobile phone auscultation

## Abstract

**Background/Objectives:** Chronic obstructive pulmonary disease (COPD) remains a condition with high morbidity, mortality, and misdiagnosis. The gold standard pulmonary function testing with spirometry has limited availability. This study seeks to test a novel diagnostic test based on auscultatory mapping of pulmonary dynamics. This NIH-funded study aimed to develop a COPD detection technology, using mobile phone auscultation, for situations in which spirometry is not available. **Methods:** This prospective study collected mobile phone auscultation data on patients presenting for spirometry and evaluation by a pulmonologist. All subjects had same-day or recent (less than 6 months) spirometry in one PFT laboratory. After informed consent, the subjects underwent respiratory auscultation using a selection of mobile phone brands. The auscultation methods included normal breathing observed at the left axillary site and egophony observed at the right supra clavicular fossa. The team created models from the recordings using Time Series Dynamics (TSD), proprietary software that uses computational nonlinear dynamics to characterize the respiratory biofluid dynamics implied by the acoustic data. **Results:** We enrolled a total of 108 patients (34.3% male), from 19 to 85 years of age (median 61 years). Among the patients, 64 (59.3%) subjects identified as White, 43 (39.8%) as Black, and 1 as Asian. Among the two cohorts with diverse comorbidities, 52 subjects had confirmed COPD and 56 did not. The cohorts differed significantly in age and body mass index, but not in race, number of comorbidities, or COPD assessment test scores. They had significant differences in forced expiratory volume in 1 s (FEV1), the FEV1/FVC (forced vital capacity) ratio, but not FVC. The recordings from the egophonic and axillary sites were initially modeled separately and then combined in a single composite model. The modeling produced excellent results with 90%+ AUC and sensitivity in both the test and train sets relative to the gold standard. **Conclusions:** Evidence suggests that a mobile phone auscultation device can accurately determine COPD diagnosis. In frontline applications where the availability of gold standard pulmonary function testing is limited, the device could improve the detection of COPD, a condition with significant over- and under-diagnosis. Future trials will investigate the ability of patients to self-record. Success would support remote COPD testing using transmitted telehealth recording data, bringing diagnosis to patients in underserved populations.

## 1. Introduction

Chronic obstructive pulmonary disease (COPD) is the most common chronic lung disease and a major cause of morbidity and mortality (the third leading cause of death worldwide) [1]. Individuals in rural areas and with low socioeconomic status are more at risk for COPD [2]. While COPD was once thought to be a uniformly progressive disease, recent studies have shown that the most rapid decline in respiratory function may occur earlier in the disease course [3]. During this crucial period, interventions such as smoking cessation and pharmacologic therapies may be most beneficial [4].

Despite the significant prevalence of COPD, there continues to be challenges with accessible, accurate, and reproducible diagnostics. Pulmonary function testing (PFT) remains the gold standard for the diagnosis of COPD but requires specialized personnel and equipment that are often located at larger, usually urban, medical centers. Furthermore, COPD has a high rate of misdiagnosis and under-diagnosis [5]; in some cases, only two-thirds of persons with spirometric evidence of COPD had a formal diagnosis [6]. Lastly, PFTs can be difficult to administer as patients must give their maximum effort, avoid pausing, coughing, or obstructing the airway, in order to achieve valid, reproducible results [7].

Alternative, accessible, and accurate modalities are needed to serve diverse, particularly marginalized, patient populations and facilitate the earlier identification of at-risk patients. Mobile phones are well suited to fill the gap. Firstly, mobile phones are practically omnipresent in contemporary society, even among the most rural and low-income populations [8]. Secondly, they possess sensitive microphones with high recording speeds that can be leveraged with novel analytical methods in the era of increasing computational power. Mobile phone auscultation could provide an alternative to spirometry, particularly for patients unable to participate in PFTs or for use in out-of-hospital settings. This study assessed the feasibility of developing predictive models for the diagnosis of spirometry-confirmed COPD using mobile phone-captured auscultation data. From our results, mobile phone auscultation (MPA) achieved impressive sensitivity, specificity, and AUC for the diagnosis of COPD. Unlike methods currently used, our method did not rely on specialized hardware, neural or spectral networks, or ultrasound.

## 2. Methods

### 2.1. Settings and Participants

This prospective, NIH-funded study collected data from a convenience sample of patients presenting to a single outpatient pulmonology clinic at an urban tertiary care medical center. Prior to enrollment, the stated aims were to reproduce the diagnosis of COPD in binary terms. We approached all patients with the intention of enrolling 50 COPD cases and 50 controls.

Inclusion criteria were the following: evaluation by a pulmonologist for respiratory disease, completed pulmonary function testing within six months (most spirometry occurring on the day of the phone recording), age over 18, or presence of spirometry interpretation by board-certified pulmonologist. Patients were excluded from participation if under 18 years of age, pregnant, or unable to perform spirometry.

The COPD group had a verified diagnosis of COPD and FEV1/FVC < 0.7. The control group had no diagnosis of COPD and an FEV1/FVC > 0.7. Some subjects in the control group were diagnosed with diseases such as asthma, sleep apnea, obesity hypoventilation syndrome, or other respiratory conditions with respective spirometry abnormalities. Enrollers approached all patients presenting for testing or office visit to the pulmonologist; thus, the patient population had a variety of respiratory symptoms.

To minimize interference, the protocol excluded patients with mechanical heart valves, along with pregnant patients and those younger than 18 years of age. The study was approved by the IRB. Patients were approached after completion of their scheduled spirometry to ensure no delays in care. The study team did not advise or perform any testing or treatment; no subjects had alterations in their care plan based on the study. All subjects signed informed consent prior to obtaining recordings or abstracting any protected health information.

### 2.2. Data Collection and Storage

**Figure 1:**
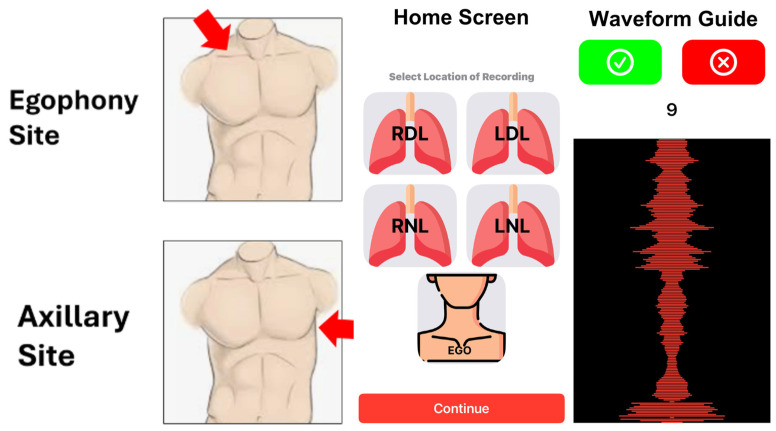
Recording sites and app screenshots.

The research team obtained demographic and clinical information from the electronic medical record. Recordings were obtained using a selection of mobile phone brands and a single recording software (in 44.1 KHS WAV format). The auscultation methods included normal breathing at the left axillary site and during egophony at the right supraclavicular fossa, a technique previously developed for detecting COVID-19 through mobile phone auscultation (see Figure 1) [9]. To recreate a stable and reproducible airflow, egophony involved the patient saying “e” for one full breath, then repeating. The axillary recording also utilized two normal breaths with no phonation. Sound recording files were uploaded into CardBox (Box Inc., Redwood City CA USA), a web-based, encrypted research cloud space. Figure 1 also shows screenshots from the sound recording app that was developed specifically for this study.

Enrollers obtained data from both patient questionnaires and the medical record. Subjects were screened with the validated COPD assessment test (CAT) [10] which quantifies the impact of the general COPD status on patients’ overall health. This information, along with demographics and other clinical data, was stored in REDCap, an online, encrypted database management software. At all times, only IRB-approved personnel had access to the stored data. The participants did not receive any compensation for participation.

### 2.3. Technology and Analytic Method

Mobile phone auscultation (MPA), classified as Class 2 software as a medical device (SaMD), can aid in the diagnosis and monitoring of patients where reliable testing is not available, for example, in the impromptu examination of telehealth patients. The mobile device comprises a recording application that allows auscultation data to be captured through standard mobile phone microphones in a consistent format across all major brands. To the user, it functions essentially like a phone voice recorder and provides a waveform guide. In published and unpublished prior work, these methods have been used to create successful MPA diagnostics for COVID diagnosis [9], heart failure/low ejection fraction [11], volume overload [12], pneumonia, and influenza.

This protocol used computational nonlinear dynamics to process mobile phone-collected auscultation data into a mapping of biofluid dynamics that can then be used to classify organ functionality. It is a physics-based approach that would not be considered artificial intelligence or machine learning under the current FDA glossary. The study subcontractor developed diagnostic algorithms using the Time Series Dynamics (TSD) modeling software, based on the recordings and certain de-identified information in REDcap. TSD modeling is a rigorous computational nonlinear dynamics approach to modeling time series observations of low-dimensional chaotic systems for the purposes of prediction or classification. When applied to auscultation data, TSD modeling is effectively a chaos-based biofluid dynamics approach. The purpose of this analysis is to set a performance benchmark for the TSD modeling approach in terms of information that could be known in impromptu examinations of remote patients (e.g., age, gender, and symptoms).

To achieve the primary aim of a binary COPD diagnostic classifier, TSD models were fitted separately to the axillary and egophony recordings and then combined into a single “composite” model. In the TSD process, recordings were mapped to 300+ phase space portraits. These portraits have been developed from a prior published and unpublished study of over 12,000 auscultation recordings. Each portrait is designed to highlight an aspect of the biofluid dynamics such as pneumodynamic resistance. The portraits were collapsed to two-dimensional space by using an entropic metaheuristic method. The method identified the most important dynamics but did not fit them to the dependent variable.

The portraits were developed from multiple prior studies using TSD modeling. The dimensions are predictors that can be used in regression models. They were not derived by principal component analysis (PCA) because PCA is limited by linear assumptions. They do, however, represent minimum entropic solutions which correspond roughly to maximum variance. When the variance is high, more information is in the tails; and the value of Shannon entropy is less because the degree of surprise is lowest.

Logistic regression was then used to fit the dependent variable to the two dimensions. Both test and train sets were evaluated in terms of AUC, sensitivity, and specificity. Comparison of the test and train set performance serves as a check against overfitting and inconsistent representation of the underlying universe. The accuracy of the model was then evaluated across various patient subgroups. Test and train sets were created by 80/20 random sampling of clustered records. The sets were based on case numbers and recordings followed. All models had the same test–train splits based on cases. At no point is knowledge of the test set used. The test data is only used to evaluate the best outputs. Phase space portraits were examined for the presence of unstable period orbits that are commonly visible in low-dimensional chaotic systems.

Patient demographics, medical co-morbidities, and pulmonary function testing were summarized using totals and proportions and stratified in order to compare those with and without COPD. Descriptive and comparative statistics were performed utilizing parametric and non-parametric testing, where appropriate based on distribution of data. All analyses were performed using STATA/IC, version 18.0 (StataCorp, College Station, TX, USA).

## 3. Results

This study met the pre-specified enrollment goals of at least 50 subjects in each of the COPD and comparator groups. From 1 December 2023 to 24 July 2024, the team enrolled a total of 108 patients (34.3% male) ranging from 19 to 85 years of age (median 61 years). Among the patients, 64 (59.3%) identified as White, 43 (39.8%) as Black, and 1 as Asian. BMI ranged from 11.6 to 65.4 kg/m^2^ (median 29.75 kg/m^2^).

Of the 108 subjects, 52 had a confirmed diagnosis of COPD based on clinical findings, imaging, and spirometry as determined by a pulmonologist. The other 56 subjects did not have COPD, but did have various other pulmonary conditions (e.g., restrictive lung disease, asthma) and general comorbidities (Table 1). See supplementary Table S1 for the classification of medical comorbidities for all subjects and stratified by subgroup.

### 3.1. COPD Diagnosis

Excluding no patient records, the team developed two models to investigate the primary aim. The TSD modeling of the egophony and axillary recordings both produced very good confusion matrix results for a binary classifier. The TSD models were initially fitted separately to the axillary and egophony recordings and then combined into a single “composite” model (Table 2). All TSD-developed models of the egophony and axillary recordings produced training sets with performance metrics above 90%. The TSD modeling of the combined auscultation observations produced excellent results with 90%+ AUC and sensitivity in both the test and train sets.

### 3.2. Subgroup Accuracy

The accuracy of the model was evaluated across various patient subgroups (Table 3). In general, the model performed well across different demographics, BMI, past medical history, and tobacco use history. The exploratory subgroup analysis, albeit with small n’s, found no drop in accuracy level below 85%.

Table 4 displays the PFT results for all subjects and subgroups. The groups differed significantly in forced expiratory volume in 1 s (FEV1) and FEV1/FVC ratio. They also differed in other spirometric parameters such as total lung capacity, residual volume, and diffusing capacity.

### 3.3. COPD Assessment Test Survey

Subjects answered questions from the COPD assessment test (CAT), to determine severity of symptoms in the subjects diagnosed with COPD, but also to compare symptoms between cohorts. Analysis showed no statistically significant differences between groups in aggregate or in any individual metric (Table 5).

### 3.4. Examination of Low-Dimensional Chaos

**Figure 2:**
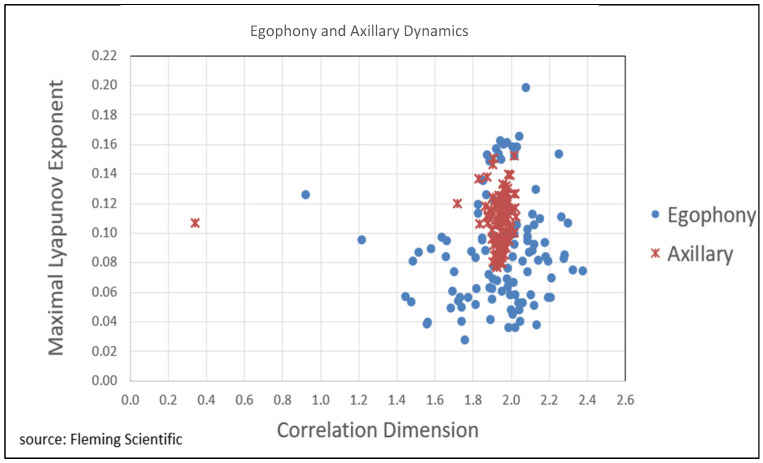
Maximal Lyapunov exponent and correlation dimensional evaluated on all recordings confirm that all are low-dimensional chaos.

An evaluation of the maximal Lyapunov exponent (MLE) and correlation dimension (Dcorr) found that all recordings are low dimensional (Dcorr < 2.5) and likely chaotic (MLE > 0) (Figure 2). One outlier was observed and confirmed by data table clustering, but not excluded. As the mean values for the egophony and axillary recordings are close, it is conceivable that these auscultations capture at least some similar dynamics despite significantly different protocols.

### 3.5. Case Description

**Figure 3:**
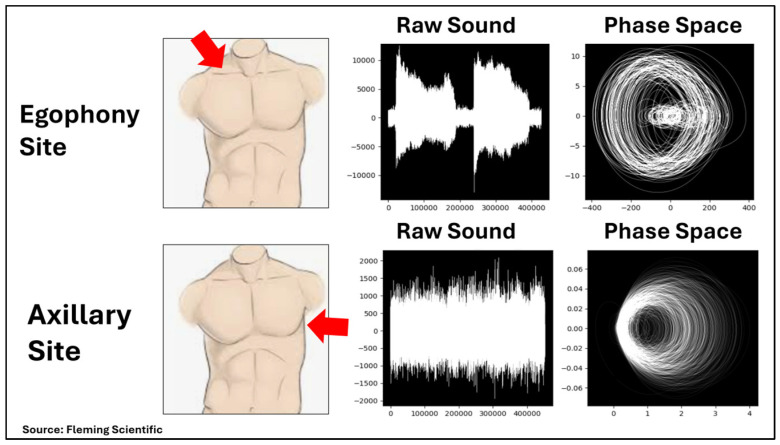
Auscultation sites and sound recording depictions.

A typical case from this study was visualized first from the egophony recording and then from the axillary recording (Figure 3). The “raw sound” waveform is displayed by the device while recording. The raw sound data is then mapped to phase spaces for analysis. Here, a phase space was created by plotting each point versus its instantaneous slope over a section of the raw sound.

Despite significant differences in the appearance of the axillary and egophony waveforms, the phase spaces were similar in the presence of unstable periodic orbits which is a common property of chaotic systems. The ability to see these orbits in only two dimensions confirms that the data is low dimensional as suggested by Dcorr.

## 4. Discussion

This study achieved the primary aim of accurate COPD diagnosis using mobile phone auscultation. In a relatively diverse cohort, with a mix of respiratory symptoms and conditions, MPA achieved notable sensitivity, specificity, and AUC for the diagnosis of COPD. The test methods did not rely on specialized hardware, neural or spectral networks, or ultrasound.

Spirometry, a standard PFT method, is commonly used to evaluate respiratory diseases. It remains the gold standard for diagnosing, staging, and monitoring COPD. Forced expiratory volume in one second (FEV1) measures the amount of air exhaled in the first second of a forced breath. Considering FEV1 to forced vital capacity (FVC) in a ratio (FEV1/FVC) can help distinguish between obstructive and restrictive diseases. In obstructive diseases, FEV1 is reduced due to increased airway resistance to expiratory flow, leading to a lower FEV1/FVC ratio [13].

The cohorts in this study had diverse results for the spirometry parameters. The COPD group had differences in FEV1 and FEV1/FVC ratio, but they did not have statistically significant differences in FVC. Thus, the MPA approach could potentially delineate cohorts who have similar FVC. COPD has high heterogeneity of both clinical symptoms and diagnostic testing [14]. Metrics that change earlier in the disease course, such as oscillometry and resting lung volumes/capacities, can provide greater sensitivity than the standard FEV1 and FEV1/FVC ratio [14].

The cohorts in this study had no significant differences in the COPD assessment test or any of its individual questions. While the chief purpose of the CAT remains to characterize patients with known COPD, this symptom overlap in COPD and non-COPD subjects remains relevant. A technology like MPA that could differentiate groups with very similar questionnaire responses could aid in both diagnosis and severity grading.

As mentioned above, spirometry requires expertise, specialized equipment, and patient cooperation to perform. It is often not available in underserved, telehealth, or emergency/first-line healthcare environments. This makes spirometry challenging to implement in numerous environments. To bring spirometry to remote settings, several portable products have been introduced for use in ambulatory settings or even nonclinical locations [15,16,17,18,19,20]. While some of these technologies pair with mobile phones (one [16] using Bluetooth), all require the use of proprietary hardware to record the spirometry air flow.

A non-invasive, cost-effective, readily available, and skill-independent test could bring respiratory diagnostics to underserved areas worldwide. Lung auscultation with mobile phones using built-in microphones represents a promising technology for monitoring respiratory conditions outside of medical facilities. This approach could also be used as a screening tool to assess individuals needing further testing for a confirmatory diagnosis. The real-time analysis of respiratory sounds’ specific signal characteristics, including biofluid dynamics, would allow clinicians to amplify, review, and compare recordings.

Our approach records subjects during inhalation and exhalation at the left axillary site and during egophony (while saying “e”) at the right supraclavicular fossa. Egophony provides a method of stabilizing airflow to standardize recordings. From the recordings, the team extracts and analyzes data to characterize biofluid dynamics (true physical sounds) without machine learning. Very little has been published using a similar approach.

One study attempted to predict spirometry results using mobile phone recordings of recorded cough sounds [21]. Their approach in 272 training subjects and 50 test subjects achieved a sensitivity and PPV of 84.95% and 98.51%, respectively. Regression models achieved root mean square error (and correlation coefficients for standard spirometry parameters FEV1, FVC, and FEV1/FVC of 0.593 L (0.810), 0.725 L (0.749), and 0.164 (0.547), respectively; this represented “high positive correlation in predicting FEV1 and FVC and moderate positive correlation in predicting FEV1/FVC” [21]. Cough sounds are highly stochastic and difficult to reproduce, partly due to recording with an open mouth. Using the phone as a stethoscope, transdermal recording eliminates ambient noise.

Irina et al. used proprietary hardware to both detect diaphragm mobility and to, then, encourage pulmonary rehabilitation by training inspiratory muscles [22]. The LASARRUS proprietary device involves a wearable multi-modal electroacoustic system that employs a sensor network with multi-modal sensing capability (e.g., digital stethoscope, electrocardiogram monitor, thermometer, and goniometer). In a pilot study with 35 healthy subjects and 3 with COPD, the group used machine learning to analyze the signal to noise ratio to detect breathing intensity [23]. While this device may be capable of auscultation, the literature thus far has not described acoustic data analysis, and the device would be subject to the same cost and logistical constraints of any other equipment.

A 2018 systematic review and meta-analysis of mobile health applications in the self- management of patients with COPD found eight studies that described mobile phone monitoring of COPD [24]. None of these applications deployed mobile phone auscultation. They instead used questionnaires, activity measuring, oxygen saturation, heart rate, respiratory rate, symptom diaries, and other non-acoustic parameters. But even these less sophisticated interventions reduced the rate of admission for COPD. Combining auscultation of physical sounds could synergistically improve the outcomes.

A scoping review of 15 studies on smartphone apps in COPD self-management found that the convenience of these apps can empower patients to be involved in their care [25]. Patients notice any worsening of symptoms and potentially detect exacerbations earlier, again preventing COPD hospitalizations. A 2023 review on publicly available mHealth apps found common uses of education, medication reminders, symptom tracking, journaling, and action planning [26].

Beyond the initial diagnosis of COPD, exacerbations lead to unscheduled medical visits, emergency department utilization, and hospitalizations. Stethoscope auscultation represents the chief means of categorizing the severity of an exacerbation. However, the conventional use of a stethoscope has limitations such as experience and the skills of the user. Additionally, sounds recorded by a traditional stethoscope cannot be saved or quantified, making retrospective analysis impossible. Furthermore, with the increasing use of telemedicine, there is no conventional alternative for the use of a stethoscope.

### Limitations

This study has several limitations. While diverse, the relatively small cohort was derived from a single institution, thus potentially not fully representative of the national distribution. Due to convenience sampling, participants may have had different COPD severity levels and, as reflected in Supplementary Table, different incidences of some comorbidities (most notably interstitial lung disease). Future work involving larger sample sizes would allow for grading of severity. Also, larger sample sizes from various institutions would allow for external validation.

As seen in baseline characteristics, a significant portion of the non-COPD subjects had restrictive lung disease. We consider this a strength rather than a limitation, as many patients undergoing evaluation for COPD could have restrictive or fibrotic disease. No definitive conclusions can be drawn from the subgroup analysis accuracies due to the small subgroup sizes. The patients did not operate the smartphones to record audio samples. Future studies will involve patients as operators, as we ultimately intend to enable patients to use this technology outside of medical facilities.

The results were evaluated by standard confusion matrix analysis for both the test and train sets. The comparison of the test and train set performance serves as a check against overfitting and the non-uniform representation of the underlying universe. In the FDA glossary, this would be considered a “tuning” step. The accuracy of the model was evaluated across various patient subsets. The only significant drop off in performance was among patients with a history of coronary artery disease (16 subjects). Otherwise, the model performed well across all subgroups. The use of frequency analysis would not have been justified.

The examination of common spectral markers, such as those from power spectrum, showed no modeling utility. The successful use of least squares, rather than neural networks, makes it possible to model much smaller data sets which could greatly enable future phase 1 research. Future studies will deploy this technology to larger populations in a phase 2 protocol taking place in remote settings, telehealth, emergency departments, and ambulatory care settings. Future trials will also need to assess validity in unsupervised settings.

## 5. Conclusions

COPD affects millions worldwide and carries significant morbidity and mortality. Previous research has demonstrated that individuals in rural and lower socioeconomic areas are at higher risk for carrying the disease and for under-diagnosis. Diagnostic tools that do not require expertise or specialized equipment could benefit patients in resource-limited areas and allow for remote monitoring by patients and healthcare providers. This study supports the idea that prescreening with mobile phone auscultation, or adding it to gold standard pulmonary function testing, could improve the diagnosis of COPD. Further trials and modeling will support the use of mobile phone auscultation in larger patient populations and by patients themselves.

## Figures and Tables

**Table 1:** Demographics and medical comorbidities for all subjects and stratified by subgroup.

Variable	Total (*n* = 108)	COPD Group (*n* = 52)	Comparison Group (*n* = 56)	*p* **
Age—years, Median (IQR)	61 (50–68.5)	63 (56–70)	57.5 (39–66.5)	0.03 *
Female sex—*n* (%)	71 (65.7)	34 (65.4)	37 (66.1)	0.94
Race—*n* (%)				
White	64 (59.3)	31 (59.6)	33 (58.9)	0.63
Black/African American	43 (39.8)	21 (40.4)	22 (39.3)	
Asian	1 (0.9)	0 (0.0)	1 (1.8)	
Ethnicity, Non-Hispanic—*n* (%)	108 (100)	52 (100)	56 (100)	NA
BMI kg/m^2^—*N*, median (IQR)	29.7 (24.5–35.5)	28.4 (23.5–31.2)	31.2 (25.1–37.5)	0.02 *
BMI Category—*n* (%)	*n* = 107	*n* = 51	*n* = 56	
<18.5	3 (2.8)	3 (5.9)	0 (0.0)	0.11
18.5–24.9	29 (27.1)	15 (29.4)	14 (25.0)	0.65
25–25.9	23 (21.5)	13 (25.5)	10 (17.9)	0.37
30–34.9	25 (23.4)	14 (27.6)	11 (19.6)	0.37
≥35.0	27 (25.2)	6 (11.8)	21 (37.5)	0.002
Medical co-morbidities				
0	4 (3.7)	1 (1.9)	3 (5.4)	0.62
1	15 (13.9)	7 (13.5)	8 (14.3)	0.90
2	23 (21.3)	13 (25.0)	10 (17.9)	0.37
3	19 (17.6)	11 (21.2)	8 (14.3)	0.35
4	11 (10.2)	5 (9.6)	6 (10.7)	0.85
5+	35 (32.4)	14 (26.9)	21 (37.5)	0.24

* Non-parametric testing performed using the Mann–Whitney rank sum test. ** Parametric testing performed using chi-squared or Fischer’s exact where appropriate based on appropriate cell size.

**Table 2:** Time series dynamics modeling of the combined recordings on COPD diagnosis.

Performance Test	Auscultation Source					
	Egophony		Axillary		Composite	
	Train	Test	Train	Test	Train	Test
Area Under the Curve	0.92	0.87	0.98	0.87	0.99	0.96
Sensitivity	0.93	0.83	0.98	0.83	1.00	0.92
Specificity	0.91	0.90	0.98	0.90	0.98	1.00

**Table 3:** TSD modeling accuracy across patient subgroups. Pulmonary function testing results.

	BMI > 30	BMI ≤ 30	Age < 50	Age ≥ 50 < 65	Age ≥ 65	Male	Female	White	Not White
*N*	53	55	24	47	37	37	71	64	44
*N*—Train	42	44	16	39	31	26	60	49	37
*N*—Test	11	11	8	8	6	11	11	15	7
Train Accuracy	100%	98%	100%	100%	97%	96%	100%	100%	97%
Test Accuracy	91%	100%	100%	100%	83%	91%	100%	93%	100%
	Hx Asthma	Hx No Asthma		Hx ILD	Hx No ILD	Hx HTN	Hx No HTN	Hx CAD	Hx No CAD
*N*	33	75		19	89	58	50	16	92
*N*—Train	25	61		16	70	44	42	13	73
*N*—Test	8	14		3	19	14	8	3	19
Train Accuracy	100%	98%		94%	100%	98%	100%	99%	99%
Test Accuracy	100%	93%		100%	95%	93%	100%	100%	100%
	Hx HLD	Hx No HLD	Hx DM2	Hx No DM2	Hx CKD/ESRD	Hx No CKD/ESRD	Hx HF	Tob Ever	Tob Never
*N*	49	59	25	83	17	91	11	80	28
*N*—Train	41	45	18	68	14	72	9	66	20
*N*—Test	8	14	7	15	3	19	2	14	8
Train Accuracy	98%	100%	100%	99%	93%	100%	100%	98%	100%
Test Accuracy	88%	100%	86%	100%	100%	95%	100%	93%	100%

Hx = History; ILD = Interstitial Lung Disease; HTN = Hypertension; CAD = Coronary Artery Disease; DM2 = Type 2 Diabetes; CKD = Chronic Kidney Disease; ESRD = End-Stage Renal Disease; HF = Heart Failure; Tob = Tobacco Treatment Measures.

**Table 4:** Pulmonary function testing, including spirometry, lung volumes, and diffusing capacity, for all subjects and by subgroup.

Variable	Total	COPD Group	Comparison Group	*p*
Spirometry				
Forced Vital Capacity (FVC)				
Pre Z-score—*n*, mean (SD)	108, −1.04 (1.37)	52, −1.24 (1.45)	56, −0.86 (1.27)	0.15
Pre—*n*, median (IQR)	108, 2.80 (2.19–3.42)	52, 2.62 (2.14–3.22)	56, 2.86 (2.31–3.78)	0.07
Pre-percent predicted—*n*, mean (SD)	108, 84.6 (20.1)	52, 81.7 (21.9)	56, 87.4 (17.9)	0.14
Post Z-score—*n*, mean (SD)	50, −0.63 (1.36)	21, −0.62 (1.58)	29, −0.64 (1.21)	0.96
Post—*n*, mean (SD)	57, 3.30 (1.13)	26, 3.11 (1.10)	31, 3.45 (1.14)	0.27
Post-percent predicted—*n*, mean (SD)	57, 90.5 (20.6)	26, 90.3 (24.6)	31, 90.6 (17.0)	0.97
Forced Expiratory Volume in one second (FEV1)				
Z-score—*n*, mean (SD)	108, −1.64 (1.60)	52, −2.43 (1.66)	56, −0.90 (1.14)	<0.0001
Pre—*n*, median (IQR)	108, 1.9 (1.40–2.47)	52, 1.40 (1.00–1.90)	56, 2.30 (1.81–2.93)	<0.0001
FEV1 percent predicted—*n*, median (IQR)	108, 0.73 (0.54–0.92)	52, 0.54 (0.40–0.71)	56, 0.85 (0.73–1.00)	<0.0001
FEV1/FVC—*n*, mean (SD)	58, 68.7 (15.4)	27, 56.30 (10.47)	31, 79.52 (9.75)	<0.0001
Lung Volumes—median (IQR)				
Total Lung Volumes (TLC = Total Lung Capacity)	*n* = 98	*n* = 47	*n* = 51	
TLC ULN	6.60 (5.88–8.24)	6.51 (5.83–8.24)	6.93 (5.97–8.37)	0.53
TLC LLN	4.46 (3.91–5.40)	4.38 (3.85–5.40)	4.69 (3.95–5.54)	0.54
TLC Z-Score	−0.54 (−1.95–0.39)	0.17 (−0.51–0.63)	−1.54 (−2.5–−0.48)	<0.0001
TLC Pre	5.19 (4.39–6.31)	5.67 (5.01–6.51)	4.70 (3.76–5.78)	0.0004
TLC Pre-percent predicted	93 (78–105)	103 (94–108)	83 (70–94)	<0.0001
Residual Volumes (RV)	*n* = 97	*n* = 47	*n* = 50	
RV ULN	2.73 (2.50–3.06)	2.78 (2.57–3.05)	2.71 (2.4–3.1)	0.5159
RV LLN	1.08 (0.97–1.28)	1.09 (1.03–1.32)	1.07 (0.79–1.28)	0.2451
RV Pre	2.13 (1.54–2.70)	2.70 (2.17–3.63)	1.65 (1.15–2.10)	<0.0001
RV Pre-percent predicted	110 (85–140)	141 (117–191)	88 (75–106)	<0.0001
Residual volume percent Total Lung volume	*n* = 97	*n* = 47	*n* = 50	
RV% TLC ULN	45 (38–49)	46 (40–48)	43 (35–50)	0.2373
RV% TLC LLN	23 (18–25)	24 (19–25)	21 (16–26)	0.2826
RV% TLC Pre	39 (31–48)	47 (41–58)	34 (28–38)	<0.0001
RV% TLC Pre-percent predicted	121 (105–151)	146 (126–172)	110 (92–121)	<0.0001
Diffusing Capacity—*n*, median (IQR)				
DLCO Z-score	98, −2.36 (−4.22–−1.09)	48, −3.15 (−4.52–−1.93)	50, −1.39 (−3.92–−0.11)	0.0058
DLCO Pre	97, 13.96 (9.00–20.16)	47, 13.05 (8.84–15.51)	50, 16.65 (9.83–23.83)	0.0115
DLCO Pre-percent predicted	98, 67 (44–84)	48, 59 (44–73)	50, 79 (44–97)	0.0103

All statistics were reported as means (standard deviations) or medians (interquartile range) based on testing for normality of the data’s distribution along with appropriate parametric (t-test) and non-parametric (rank sum) testing for differences between subgroups. ULN—upper level of normal; LLN—lower level of normal.

**Table 5:** Descriptive breakdown of COPD assessment test by question and severity category for all subjects and subgroups—median (IQR).

Variable	Total *n* = 108	COPD *n* = 52	Control *n* = 56	*p*
Symptoms				
Cough	3 (1.5–4)	3 (1.5–4)	2 (1.5–4)	0.6889
Phlegm	2 (0.5–3.0)	2.5 (1–4)	2 (0–3)	0.1706
Breathlessness	3 (2–5)	4 (2–5)	3 (2–5)	0.6273
Chest tightness	1 (0–3)	1 (0–3)	1 (0–3)	0.7245
Activities	3 (0–4)	3 (1–4)	2 (0–3.5)	0.1743
Confidence	0 (0–1.5)	0 (0–2)	0 (0–1)	0.5134
Sleep	2 (0–3)	2 (0–3.5)	2 (0–3)	0.6216
Energy	3 (2–4)	3 (2–4)	3 (2–3.5)	0.3302
CAT Score Total	16 (11.5–25)	17 (12–26)	16 (10–23.5)	0.2883
Health Impact Category—*n* (%)				
Low	25 (23.1)	9 (17.3)	16 (28.6)	0.17
Medium	45 (41.7)	22 (42.3)	23 (41.1)	0.90
High	30 (27.8)	17 (32.7)	13 (23.2)	0.27
Very High	8 (7.4)	4 (7.7)	4 (7.1)	0.91

All statistics were reported as medians (interquartile range) based on testing for normality of the data’s distribution along with appropriate non-parametric (rank sum) testing for differences between subgroups. Health categorical data summarized as totals and percentages and differences between groups were assessed using chi-squared testing.

## Data Availability

The original contributions presented in the study are included in the article/Supplementary Material, further inquiries can be directed to the corresponding author.

## Supplementary Materials

The following supporting information can be downloaded at: https://www.mdpi.com/article/10.3390/diagnostics15121550/s1, Table S1: Medical comorbidities for all subjects and stratified by sub-group.
