# Supplementary material for: Mobile Phone Auscultation Accurately Diagnoses Chronic Obstructive Pulmonary Disease Using Nonlinear Respiratory Biofluid Dynamics
Source: Diagnostics (Basel). 2025 Jun 18;15(12):1550. doi: 10.3390/diagnostics15121550 (PMC12192240; doi:10.3390/diagnostics15121550)
Supplement: Supplementary file 1 [file diagnostics-15-01550-s001.zip › diagnostics-3626078-supplementary.pdf]

Supplementary Table S1. Medical comorbidities for all subjects and stratified by sub-group				
Condition	Total (N=108)	COPD Group (n=52)	Comparison group (n=56)	P**
Asthma	33 (30.6)	15 (28.9)	18 (32.1)	0.71
Bronchiectasis	4 (3.7)	2 (3.9)	2 (3.6)	0.94
Interstitial Lung Disease	19 (17.6)	1 (1.9)	18 (32.1)	**< 0.001**
Prior Lung Disease	11 (10.2)	3 (5.8)	8 (14.3)	0.21
Atrial fibrillation	7 (6.5)	4 (7.7)	3 (5.4)	0.71
Hypertension	58 (53.7)	29 (55.8)	29 (51.8)	0.69
Coronary artery disease	16 (14.8)	9 (17.3)	7 (12.5)	0.48
Hyperlipidemia	49 (45.4)	26 (56.0)	23 (41.1)	0.35
Cerebral vascular accident	8 (7.4)	6 (11.5)	2 (3.6)	0.11
Diabetes (type I)	1 (0.9)	0 (0.0)	1 (1.8)	1.00
Diabetes (type II)	25 (23.2)	10 (19.2)	15 (26.8)	0.35
Chronic kidney disease	17 (15.7)	7 (13.5)	10 (17.9)	0.53
Cirrhosis	2 (1.9)	1 (1.9)	1 (1.8)	1.00
Human Immunodeficiency Virus infection	4 (3.7)	2 (3.9)	2 (3.6)	1.00
Current anticoagulant use	12 (11.1)	4 (7.7)	8 (14.3)	0.36
Smoking history	61 (56.5)	35 (67.3)	26 (46.4)	**0.03**
Heart failure	11 (10.2)	6 (11.5)	5 (8.9)	0.76
Valvular disease^	6 (5.6)	3 (5.8)	3 (5.4)	1.00
Peripheral vascular disease	3 (2.8)	1 (1.9)	2 (3.6)	1.00
Pulmonary hypertension	8 (7.4)	5 (9.6)	3 (5.4)	0.48
Pulmonary embolism	4 (3.7)	2 (3.9)	2 (3.6)	1.00
Other$	31 (28.7)	8 (15.4)	23 (41.1)	**0.003**
Primary Lung cancer	2 (1.9)	1 (1.9)	1 (1.8)	1.00
Prior lung surgery#	1 (0.9)	0 (0.0)	1 (1.8)	1.00

** Chi2 testing versus Fisher's exact based on cell size
^ moderate to severe disease only
$ Supraventricular tachycardia, sarcoidosis (5), peripartum cardiomyopathy, severe OSA, SLE, alcohol use disorder, scleroderma (3), TBI, POTS, wegner's, cerebral aneurysm, ganglioblastoma of childhood, ankylosing spondylitis, aortic aneurysm
# lobectomy, pneumonectomy, etc.

Supplementary Table S1: The control group had diagnoses of asthma, sleep apnea, primary fibrosis, obesity hypoventilation syndrome, or other co-morbidities affecting their pulmonary function. The control group had a significantly different number of subjects with interstitial lung disease, likely driving the FVC difference the reviewer points out.
